# Formaldehyde fixation is detrimental to actin cables in glucose-depleted *S. cerevisiae* cells

**DOI:** 10.15698/mic2016.05.499

**Published:** 2016-04-12

**Authors:** Pavla Vasicova, Mark Rinnerthaler, Danusa Haskova, Lenka Novakova, Ivana Malcova, Michael Breitenbach, Jiri Hasek

**Affiliations:** 1 Laboratory of Cell Reproduction, Institute of Microbiology of the CAS, v.v.i., Prague, Czech Republic.; 2 Department of Cell Biology, Division of Genetics, University of Salzburg, Salzburg, Austria.

**Keywords:** Abp140-GFP, Abp1-RFP, Actin cables, Actin patches, yeast

## Abstract

Actin filaments form cortical patches and emanating cables in fermenting cells of *Saccharomyces cerevisiae*. This pattern has been shown to be depolarized in glucose-depleted cells after formaldehyde fixation and staining with rhodamine-tagged phalloidin. Loss of actin cables in mother cells was remarkable. Here we extend our knowledge on actin in live glucose-depleted cells co-expressing the marker of actin patches (Abp1-RFP) with the marker of actin cables (Abp140-GFP). Glucose depletion resulted in appearance of actin patches also in mother cells. However, even after 80 min of glucose deprivation these cells showed a clear network of actin cables labeled with Abp140-GFP in contrast to previously published data. In live cells with a mitochondrial dysfunction (rho^0^ cells), glucose depletion resulted in almost immediate appearance of Abp140-GFP foci partially overlapping with Abp1-RFP patches in mother cells. Residual actin cables were clustered in patch-associated bundles. A similar overlapping “patchy” pattern of both actin markers was observed upon treatment of glucose-deprived rho^+^ cells with FCCP (the inhibitor of oxidative phosphorylation) and upon treatment with formaldehyde. While the formaldehyde-targeted process stays unknown, our results indicate that published data on yeast actin cytoskeleton obtained from glucose-depleted cells after fixation should be considered with caution.

## INTRODUCTION

The actin cytoskeleton has extensively been studied in various types of eukaryotic cells. It plays a key role in various essential processes including cellular movement, protein trafficking and secretion, cell division, cell growth and plasma membrane remodeling. In the budding yeast *Saccharomyces cerevisiae*, the actin cytoskeleton is primarily composed of two morphologically distinct structures: cortical actin patches and actin cables 1. Patches are formed by actin filaments surrounding finger-like invaginations of the plasma membrane 2; the actin cables are long bundles of F-actin filaments extending from the growing bud into the mother cell. In cytokinesis and septum formation, actin filaments are arranged in a circumferential actomyosin ring at the mother-bud neck 3. Changes in F-actin distribution have also been described during mating 4, sporulation 5, aging 6, and in response to various environmental stresses, including heat shock 7, osmotic stress 8 and glucose deprivation 9. Recently, the formation of actin chunks 10 or actin bodies 11,12 has been observed in starving stationary or quiescent yeast cells. The initial information on actin structures in *S. cerevisiae* came from the studies of formaldehyde-fixed cells labeled with fluorescently-tagged phalloidin (e.g. rhodamine-phalloidin) 1 that specifically binds to F-actin 13.

To visualize actin structures in living yeast cells, engaging the fluorescent protein technology provides a distinct advantage. However, all of the GFP fusions of the yeast actin created so far were observed in patches only, and none complemented the *act1* null mutant. It was suggested that these fusions are not incorporated into actin cables 14. Similarly, the GFP fusion of the actin filament bundling protein Sac6, a protein that is specific for both cables and patches 15, was not localized to cables 14. Therefore, fluorescent fusions of other actin binding proteins were employed to visualize actin structures in live yeast cells. One of them, the actin-binding protein Abp1, has a function in endocytosis and is accumulated in cortical patches only 16. In contrast, the actin-binding protein Abp140 associates primarily with F-actin cables 17, and it has been successfully used to analyze the dynamics of the actin cytoskeleton in live *S. cerevisiae* cells 18,19. These days there is a number of live imaging microscopy studies on F-actin in fermenting *S. cerevisiae* cells (e.g. 14,19,20,21,22,23 and in stationary phase cells 11,24. At the moment there is only one recent report on actin in live glucose-depleted yeast cells 25.

In this study, we demonstrate that live rho^+^ (respiring) wild type cells depleted for glucose for 80 minutes still display a complex network of actin cables (marker Abp140-GFP) and depolarized pattern of actin patches (marker Abp1-RFP). When the cells were treated with the mitochondrial uncoupler FCCP (carbonylcyanide p-trifluoromethoxyphenylhydrazone) simultaneously with glucose deprivation, the population consisted of a large number of cells with destabilized actin cables visualized by Abp140-GFP. Several accumulations of Abp140-GFP partially overlapped with the Abp1-RFP signal in cortical patches. A similar pattern was found in glucose-deprived rho^+^ cells after formaldehyde fixation and in live glucose-deprived cells with mitochondrial dysfunction (rho^0^ cells). We assume that stability of actin cables reflects the metabolic status of the cell. Based on comparison of live and formaldehyde-fixed cells, our data suggest that formaldehyde affects respiration before fixation and this uneven signaling results in destabilization of actin cables in glucose-deprived cells.

## RESULTS

### Glucose-depleted formaldehyde-fixed cells show a depolarized pattern of F-actin.

**Figure 1:**
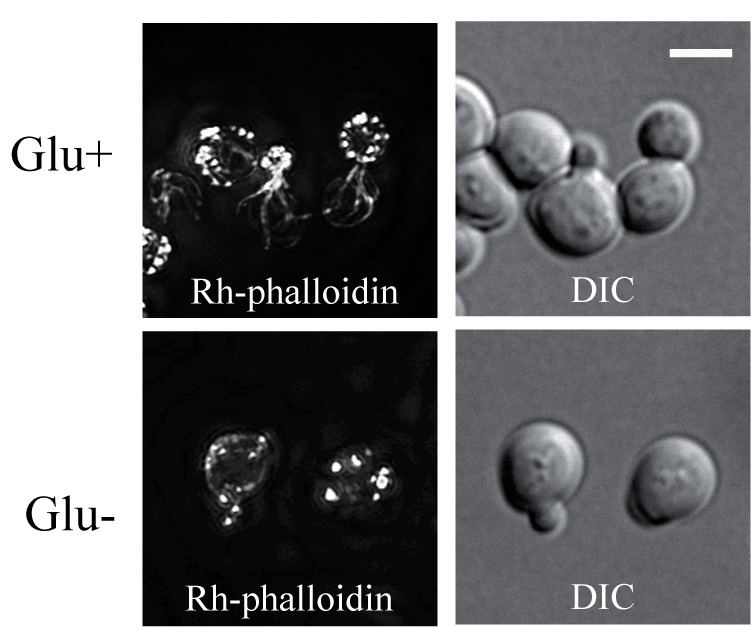
FIGURE 1: The F-actin stained with rhodamine-tagged phalloidin in exponentially growing *S. cerevisiae* (rho+) cells (strain CRY339; Z-stack). Cells were fixed with 3.7 % formaldehyde in the presence of glucose (HCHO-fixed) (Glu+), or after 30 minutes incubation in medium without glucose (Glu-). Bar, 5 µm.

Glucose-depleted *S. cerevisiae* cells arrest translation and after formaldehyde fixation display a depolarized F-actin distribution pattern labelled with rhodamine-tagged phalloidin 9. Using staining with rhodamine-tagged phalloidin (Rh-phalloidin) we confirmed these data. After fixation rho^+^ (respiring) cells that were grown in a glucose rich medium displayed polarized distribution of actin patches, usually localized to the cell cortex of daughter cells and actin cables emanating into mother cells (Fig.1 Glu+). The F-actin distribution pattern was completely different in cells starved for glucose for 30 minutes and subsequently fixed with formaldehyde in the absence of glucose. In these cells, only a depolarized pattern of F-actin chunks/accumulations labeled with Rh-phalloidin was observed (Fig.1 Glu-).

### Live glucose-depleted cells display a developed network of actin cables.

**Figure 2:**
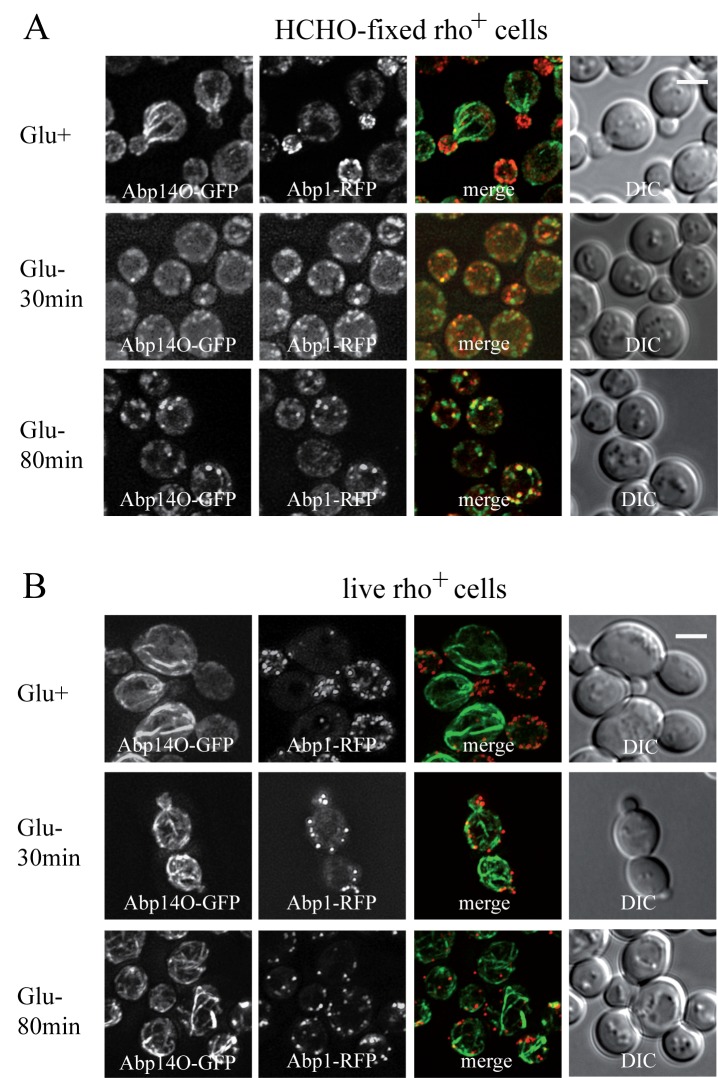
FIGURE 2: *S. cerevisiae* (rho^+^) cells co-expressing Abp1-RFP and Abp140-GFP from chromosomal sites (strain CRY1337). They were inspected after fixation with 3.7 % formaldehyde for 30 minutes (HCHO-fixed) **(A)** or as live cells **(B)**. (Glu+) glucose was present in the medium; (Glu-) cells were shifted to glucose-free medium and cultivated for an additional 30 or 80 minutes before fixation or inspection. Distribution of fluorescent markers is presented after deconvolution and projection of several images in the stack (Z-stacks) using Xcellence software (Olympus). Bar, 5 µm.

**Figure 3:**
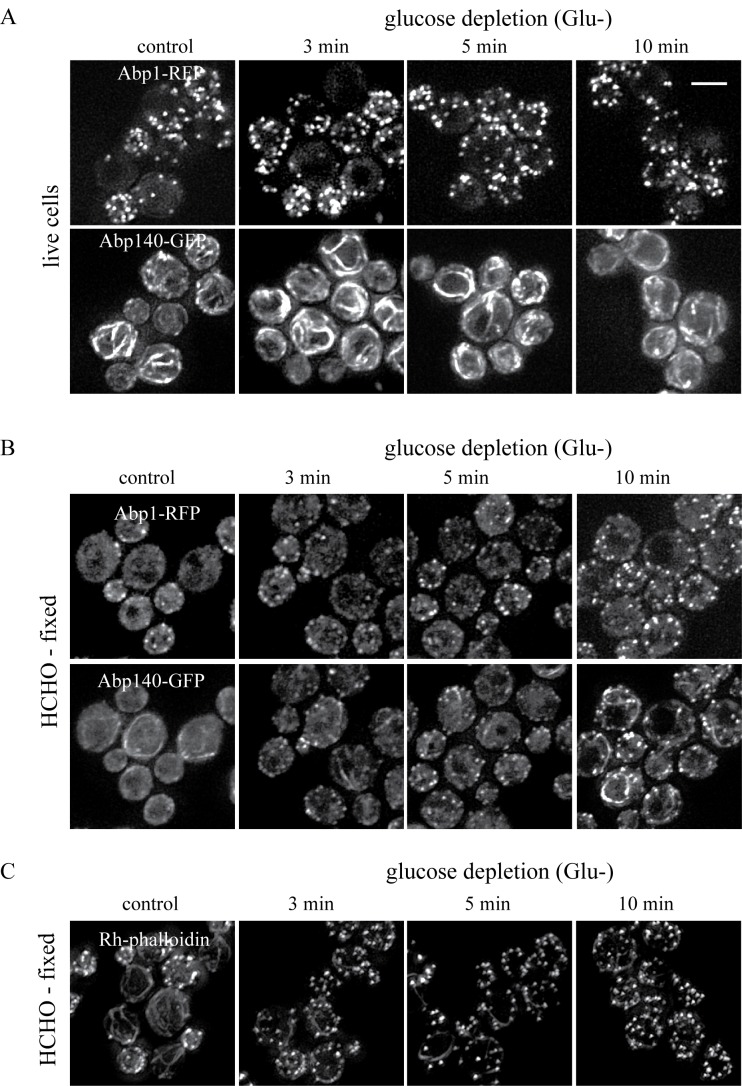
FIGURE 3: The effect of glucose deprivation on F-actin distribution was analyzed at defined time points after the shift of cells into glucose-free medium. Z-stacks of *S. cerevisiae* (rho^+^) cells co-expressing Abp1-RFP and Abp140-GFP from chromosomal sites (strain CRY1337) inspected as live cells **(A)** or after fixation with 3.7 % formaldehyde for 30 minutes **(B)** (HCHO-fixed). **(C)** The Z-stack of formaldehyde-fixed *S. cerevisiae* (rho^+^) cells (strain CRY339) stained with Rh-phalloidin for F-actin. Bar, 5 µm.

To compare our findings with published data on actin distribution in fixed 9 and live glucose-starving cells 24,25 we employed image analyses of wild-type rho^+^ (respiring) cells expressing established fluorescence markers of the two different F-actin structures patches and cables (Abp1-RFP and Abp140-GFP). Both the glucose-grown and the glucose-depleted cells were fixed with 3.7 % formaldehyde for 30 minutes, and changes in distribution of both markers Abp1-RFP and Abp140-GFP were analyzed (Fig. 2A). The pattern of actin cables (Abp140-GFP) and actin patches (Abp1-RFP) was not affected when the cells were fixed in the presence of glucose (Fig.2A, Glu+), but the filamentous pattern of Abp140-GFP and polarized distribution of Abp1-RFP almost dissipated in the cells starved for glucose for 30 minutes prior to fixation (Fig.2A, Glu- 30 min). The fluorescence signal of Abp140-GFP was accumulated in small dots. Prolonged glucose starvation up to 80 minutes resulted in appearance of chunks of both F-actin markers (Fig.2A, Glu- 80min) In contrast, our experiments on live glucose-depleted rho^+^ cells expressing both actin markers (Abp140-GFP and Abp1-RFP) revealed different actin pattern compared to formaldehyde-fixed rho^+^ cells. As expected, Abp1-RFP was localized to actin patches accumulated in buds, and Abp140-GFP labelled the actin cables emanating from the buds in cells exponentially growing in high glucose medium (Fig.2B, Glu+). A 30-minute- or a prolonged 80-minute- glucose deprivation led to a changed distribution pattern of actin patches and led to the appearance of patches also in mother cells (Fig.2B, Glu-). Whereas the polarized pattern of actin patches was lost, we did not observe any obvious loss of the actin cable integrity in these cells. These cells still display bundles of actin cables. The pattern of F-actin cables destabilization shape was studied in detailed time course glucose deprivation (Fig.3). Whereas live glucose-depleted cells displayed actin cables (Fig.3 A), glucose deprivation for 10 minutes was critical for the stability of actin cables in formaldehyde-fixed glucose-depleted cells (Fig.3 B), including those labeled with Rh-phalloidin (Fig.3 C).

We conclude that a glucose depletion up to 80 minutes does not lead to loss of actin cables and that formaldehyde fixation affects distribution of actin cytoskeleton in these cells.

### Formaldehyde fixation affects mitochondrial network in glucose-depleted rho^+^ cells.

**Figure 4:**
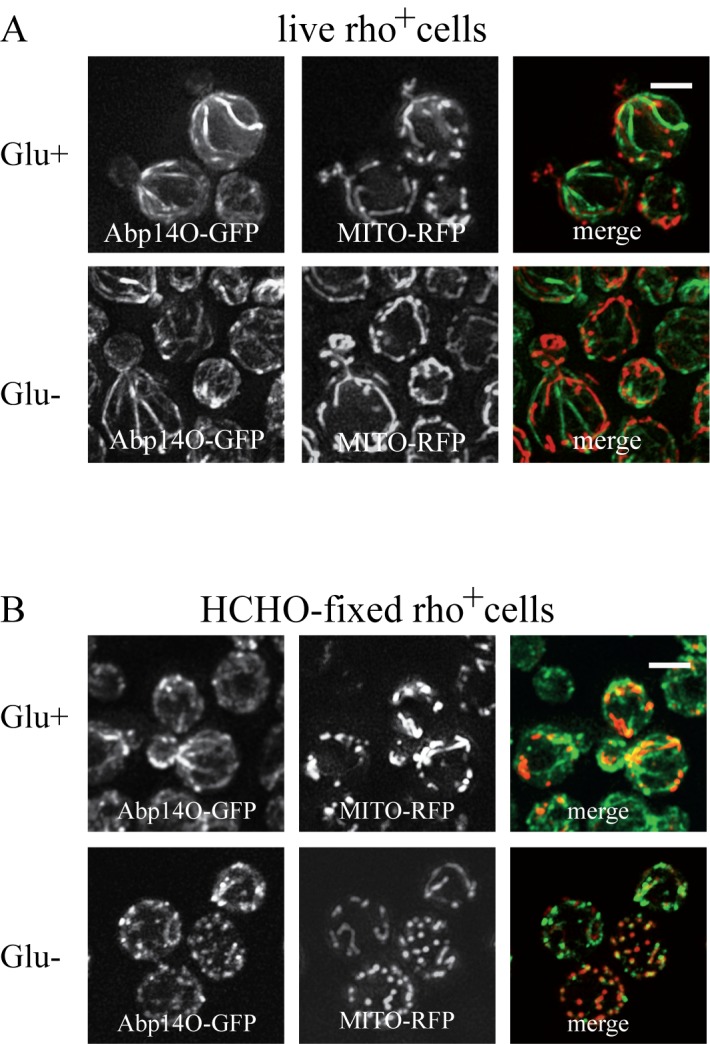
FIGURE 4: *S. cerevisiae* (rho^+^) cells co-expressing Abp140-GFP and the mitochondrial marker MITO-RFP (strain CRY816). Exponentially growing cells were inspected as live cells **(A)** or after fixation with 3.7 % formaldehyde for 30 minutes **(B)** (HCHO-fixed). (Glu+) glucose was present in the medium; (Glu-) exponentially growing cells were shifted to glucose-free medium and incubated for 30 minutes before inspection or fixation. Distribution of fluorescent markers is presented after deconvolution and projection of several image layers in the stack (Z-stack) using Xcellence software (Olympus). Bar, 5 µm.

The effect of formaldehyde fixation on the mitochondrial network was examined in strains co-expressing Abp140-GFP from the chromosomal site with the plasmid-derived RFP-tagged mitochondrial marker MITO-RFP (plasmid pYX142-mtRFPm). In live cells grown on glucose both, actin cables and mitochondria, were intact (Fig.4A, Glu+) and in glucose-deprived live cells the mitochondrial network was even more branched and tubular (Fig.4A, Glu-). In formaldehyde-fixed fermenting cells (Fig.4B, Glu+), Abp140-GFP-labeled actin cables were preserved and the mitochondrial network was not obviously altered compared to live cells. In contrast and consistent with our previous data, the formaldehyde fixation of glucose-depleted cells resulted in destabilization of both, actin cables and the mitochondrial network (Fig.4B, Glu-).

Thus, as shown by live imaging analyses, glucose starvation by itself did not affect the integrity of either the cable pattern or the mitochondrial network in live cells. However, we showed here that the combined effect of glucose starvation and formaldehyde fixation resulted in destabilization of both, the mitochondrial network and actin cables. Hence, it can be speculated, that formaldehyde fixation affects integrity and/or function of mitochondria and consequently, the integrity of actin cables is affected.

**Figure 5:**
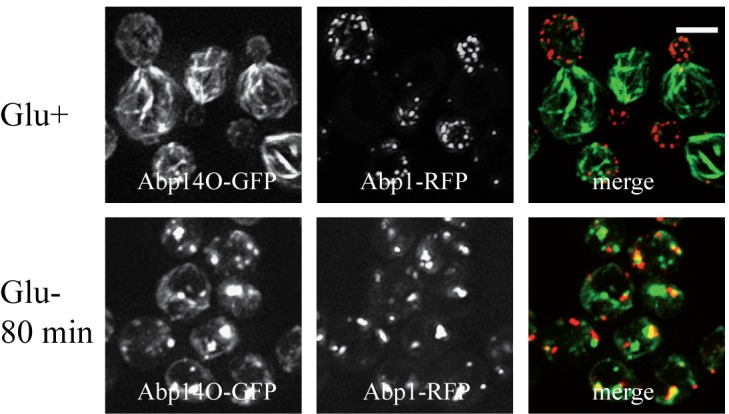
FIGURE 5: *S. cerevisiae* (rho^0^) cells co-expressing Abp1-RFP and Abp140-GFP from chromosomal sites (strain CRY1454). Exponentially growing cells were inspected directly in the presence of glucose (Glu+) or after 80 minutes glucose-deprivation in the medium without glucose (Glu-). Distribution of fluorescent markers is presented after deconvolution and projection of several images in the stack (Z-stack) using Xcellence software (Olympus). Bar, 5 µm.

### Glucose deprivation induces clustering of actin patches and loss of actin cables in respiratory deficient (rho^0^) cells.

**Figure 6:**
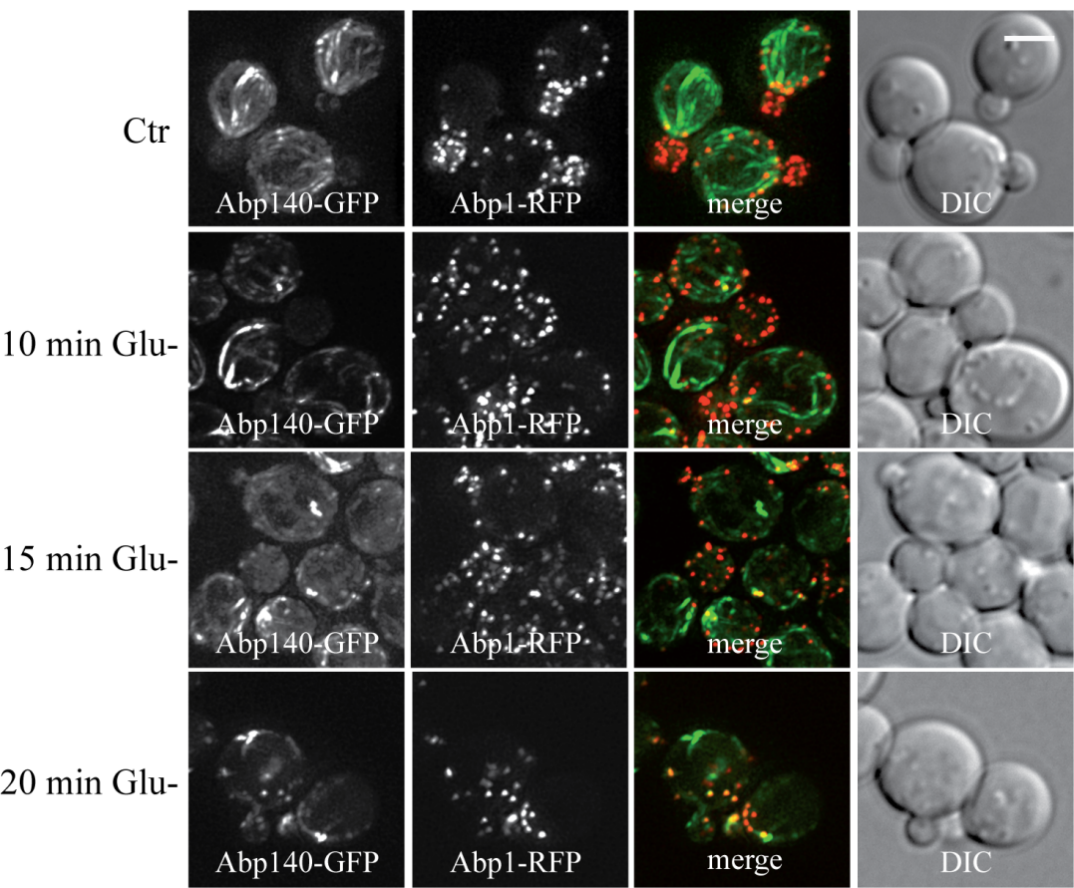
FIGURE 6: Detailed time course analyses of the glucose-depletion effect on *S. cerevisiae* (rho^0^) live cells co-expressing Abp1-RFP and Abp140-GFP from chromosomal sites (strain CRY1454). The number of Abp140-GFP filaments decreased with time of glucose depletion. Distribution of fluorescent markers is presented after deconvolution and projection of several images in the stack (Z-stack) using Xcellence software (Olympus). Bar, 5 µm.

We speculate that the effect of formaldehyde fixation on the actin cable integrity in glucose-deprived cells might be a consequence of mitochondrial dysfunction. Therefore live-cell imaging analyses were performed with respiratory-deficient ethidium bromide-induced rho^0^ cells co-expressing Abp140-GFP (actin cables) and Abp1-RFP (actin patches) from chromosomal sites. These exponentially growing rho^0^ cells displayed the polarized actin pattern consisting of cortical actin patches accumulated in buds (Abp1-RFP) and associated actin cables (Abp140-GFP) emanating into the mother cells (Fig. 5, Glu+). In contrast to rho^+^ (wild type) cells (see Fig.2B), glucose starvation of rho^0^ cells for 80 minutes resulted in an obvious loss of actin cables (Fig. 5 Glu-). Abp1-RFP was re-localized from buds also into the mother cells and both actin markers, Abp140-GFP and Abp1-RFP, were accumulated in enlarged and partially overlapping chunks or bodies. Detailed time course analyses revealed that destabilized actin cables appeared in most of the rho^0^ cells in the population after a 15 minute glucose deprivation (Fig.6).

### FCCP affects actin in glucose-depleted cells.

**Figure 7:**
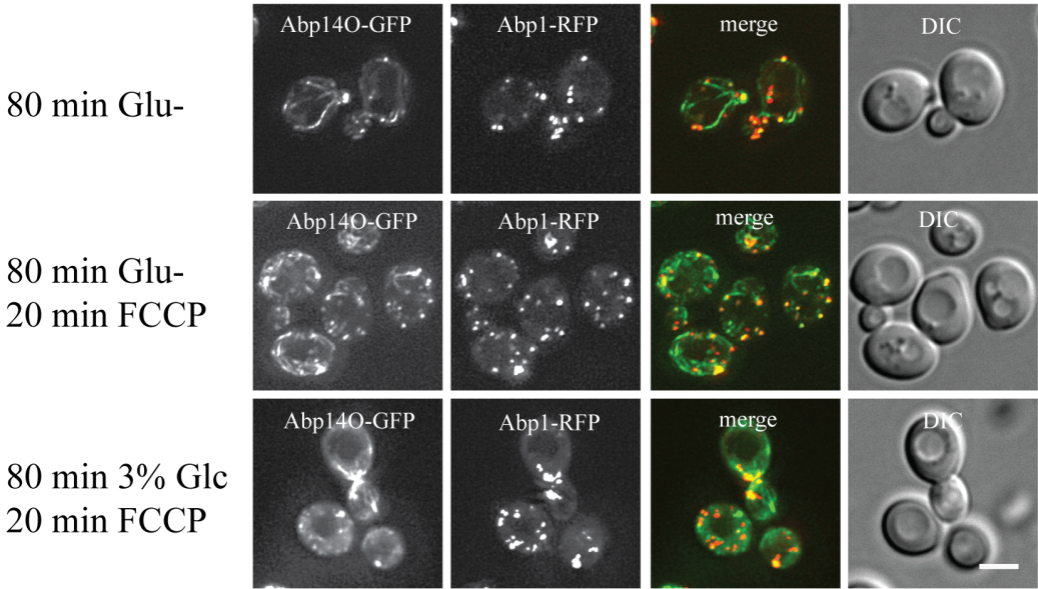
FIGURE 7: The effect of proton-ionophore FCCP on *S. cerevisiae* (rho^+^) cells co-expressing Abp140-GFP and Abp1-RFP (strain CRY1337). The live cells were either glucose-depleted for 80 minutes (80 min Glu-) or treated with 20 mM FCCP for 20 min after glucose depletion for 80 min (80min Glu-; 20 min FCCP). For comparison the cells cultivated in 3 % YPG (3 % glycerol) for 80 min were also subsequently treated with 20 mM FCCP. Treatment with FCCP generated loss of actin cables in glucose-free media. Distribution of fluorescent markers is presented after deconvolution and projection of several image layers in the stack (Z-stack) using Xcellence software (Olympus). Bar, 5 µm.

To confirm our previous data that changes in mitochondrial respiration affect the stability of actin cables in glucose-depleted cells we treated rho^+^ cells co-expressing Abp140-GFP and Abp1-RFP with the proton-ionophore FCCP (Fig. 7). The drug was added to cultures of exponentially growing cells already cultivated in either glucose-free or 3 % glycerol-containing media for 80 minutes. After a 20 minutes treatment with FCCP, most of the cells displayed only chunks of accumulated Abp140-GFP overlapping with chunks of Abp1-RFP. We conclude that mitochondrial dysfunction affects the integrity of actin cables in glucose-depleted cells.

## DISCUSSION

Microscopic analyses of the actin cytoskeleton based on the Rh-phalloidin staining of formaldehyde-fixed *S. cerevisiae* cells 1 significantly helped to understand general aspects of actin cytoskeleton organization and dynamics in eukaryotic cells. Here we present evidence, that the formaldehyde treatment induces clustering of actin patches and disorganizes actin cables in glucose-depleted cells. However, if actin cables and patches are visualized in live cells using Abp140-GFP and Abp1-RFP, respectively, these cells display also actin cables. This is in apparent contradiction to the F-actin pattern reported previously 9. 

It is evident that dynamics of actin is directly linked to growth and life cycle controls 26. In exponentially growing cells, actin patches reflect the sites of endocytosis 21, actin cables provide the track for directed transport processes like it is observed for autophagy 27 and the actomyosin ring is involved in cytokinesis 28. In contrast to exponentially growing yeast cells, the loss of both actin cables and the polarized distribution of actin patches was reported in the post-diauxic growth phase stained for F-actin with Rh-phalloidin 10,12. The fixed stationary or quiescent yeast cells display large actin accumulations named "actin bodies" 11,12. Similar actin structures were reported in live stationary yeast cells that were not able to respire 24. Interestingly, beside the cells with actin accumulations, the subpopulation of stationary yeast cells with dynamic actin cytoskeleton was also detected. The stationary cells with dynamic actin cytoskeleton revealed activated autophagy, endocytosis and well developed mitochondrial network. It is important to stress that subpopulation with intact actin cables was not observed in cells fixed with formaldehyde 11,12,24 indicating that fixation with formaldehyde might cause detrimental changes in actin cytoskeleton structure in stationary cells 24. The possibility that formaldehyde fixation may alter, under some metabolic program, the actin cytoskeleton structure has been indicated recently by Xu *et al.* 25. These authors show presence of actin cables labeled with Abp140-GFP in live glucose depleted cell, although in formaldehyde fixed glucose-depleted cells the disorganization of actin cables has been previously described by Uesono *et al.* 9. All these contradictions between actin shape in live and fixed cells support our comparison of the actin patterns in the live glucose-deprived cells and in the fixed ones that shows the accumulation of actin patches as consequence of formaldehyde treatment in the absence of glucose in the medium. In these cells, formaldehyde destabilizes actin cables and finally, both F-actin markers co-localize in enlarged "actin chunks or bodies".

A remarkable loss of actin cables in formaldehyde-fixed cells has been usually interpreted as a consequence of various stresses including osmotic stress 29, heat shock 7, glucose depletion 9, oxidative stress 23. In addition, mutations in various genes like *mdm20*Δ 30, *tpm1*Δ 31 and *whi2*Δ 32, or alterations of the translation elongation factor eEF1A 33 and the formin-based F-actin nucleation 34 were also considered to induce a loss of actin cables detected by phalloidin in formaldehyde-fixed cells. As both Abp1-RFP and Abp140-GFP were observed in enlarged chunks in live glucose-deprived rho^0^ cells (see Fig.3 Glu-), the pattern of F-actin distribution in formaldehyde-fixed cells should be viewed with caution. 

As we document here, the formaldehyde fixation affects distribution of both, actin structures and the mitochondrial network in the absence of glucose. This close interconnection between intact functional mitochondria and F-actin cables is further supported by our observations in the absence of glucose that in cells with compromised mitochondria (rho^0^) F-actin patches as well as F-actin cables are immediately collapsed. In this respect, our data are in consistence with previous conclusions that respiration is needed for actin repolarization 9,10. Recently the links between cofilin and mitochondria have been described 35. The authors clearly showed that several cofilin mutants had serious problems with mitochondrial respiration resulting in earlier formation of actin bodies. They concluded that this F-actin phenotype is related to the failure to control the *ras* signaling and they suggested that the cell death occurs within colonies formed from these cofilin mutants. However, it could be suggested that a similar failure of *ras* signaling may happen only as a consequence of the formaldehyde fixation in the absence of glucose. This still has to be elucidated. 

It is obvious that the actin cytoskeleton is directly linked to mitochondria and regulation of energy metabolism including respiration. For example, the myosin-related motor protein Myo2 has been shown recently as an essential and a direct mediator of the bud-directed mitochondrial movement in yeast 36. Since many microscopic studies on the actin cytoskeleton have been performed on formaldehyde-fixed cells, it remains unclear what the leading force in this relationship is. We assume that it is still an open question and the re-evaluation of some previously published data on the yeast F-actin rearrangement is desirable.

The fixation of yeast cells with formaldehyde or commercially available formalin according to standard protocols does not "freeze" the momentary state of the cell, but rather can induce physiological changes before killing the cell, as shown by changes in the localization of transcription factors, like Gat1 (GATA factor) 37. Similarly, there are indications from mammalian cells that formaldehyde affects calcium channels 38. In addition, in mammalian cells calcium signaling is controlled by mitochondria to coordinate energy production and consumption within cells 39. Based on the phenotype similarity with clustered Abp1-RFP patches observed in the respiratory deficient rho^0^ cells incubated without glucose, a sudden energy depletion in formaldehyde-fixed cells could be suggested to be at the origin of actin aggregation. Such irreversible ATP depletion caused by formaldehyde treatment has already been referred for human red cells 40. 

A question remains whether this detrimental effect of formaldehyde could be prevented. Details remain to be elucidated. However, based on our experience from our earlier F-actin studies in fungi 4,41,42,43,44,45, the occurrence of actin cables after formaldehyde-fixation could be stabilized by a short pre-incubation of cells with 30 mM EGTA before fixation. This suggests that formaldehyde before fixation may induce some uneven and general permeablization of intracellular membranes, thus significantly changing the intracellular homeostasis.

## MATERIALS AND METHODS

### Strains, plasmids, media and general methods

*S. cerevisiae* strains used in this study are listed in Table 1. Yeast cultures were grown in YPD medium (1 % yeast extract, 2 % peptone, 2 % glucose) or SC medium (0.17 % YNB without amino acids and ammonium sulfate, 0.5 % ammonium sulfate or 0.1 % monosodium glutamate, 2 % glucose, supplemented with a complete or an appropriate mixture of amino acids) at 30°C. Corresponding solid media contained 2 % agar. Standard methods were used for all DNA manipulations 46. The specific strains and mutants expressing GFP/RFP fusions from the sites on the chromosomes were generated by mating, subsequent sporulation in a liquid Fowell medium, and spore dissection using the Singer_TM_ micromanipulator. For glucose deprivation, exponentially growing cells were transferred to glucose-free synthetic (SC) medium and incubated under shaking for additional 30-80 min. The rho^0^ strain was prepared by the treatment with ethidium bromide as described elsewhere 47. The FCCP (Carbonyl cyanide 4-(trifluoromethoxy) phenylhydrazone; Sigma) was applied from the 10 mM stock solution in DMSO to the final 20 µM concentration. 

**Table 1:** Yeast strains.

**Strain**	**Genotype**	**Source or reference**
CRY198	*MAT*α *his3-Δ200 ura3-52 leu2-3,112 ABP1-RFP::HIS3, *rho^+^	48
CRY339	*MAT*a *his3* Δ200 *leu*2-3112 *ura*3-52 *trp1*-Δ901 *lys*2-801 *suc2*Δ9 *ABP140-GFP::KanMX, *rho^+^	This study
CRY1269	*MAT*a; CRY339; rho^0^	This study
CRY816	*MAT*a; CRY339; rho^+^; pYX142-mtRFPm	This study
CRY1337	*MAT*a; CRY339 x CRY198; *ABP1-RFP::HIS3 ABP140-GFP::KanMX; *rho^+^	This study
CRY1454	*MAT*a; CRY339 x CRY198; *ABP1-RFP::HIS3 ABP140-GFP::KanMX;* rho^0^	This study
CRY1626	*MAT*a; CRY1269; rho^0^; pYX142-mtRFPm	This study

Cells grown in presence of glucose and glucose-depleted yeast cells were fixed with formaldehyde (final concentration 3.7 %) added to the medium for 30 minutes and stained for F-actin with rhodamine-tagged phalloidin (Rh-phalloidin; Molecular Probes) as described elsewhere 1,45. The cells were inspected after coating with a slice of 1.5 % agarose in the appropriate medium as described elsewhere 49. Distribution of GFP and RFP fusion proteins was observed with a 100x PlanApochromat objective (NA=1.4) using the Olympus IX-81 inverted microscope equipped with Hammamatsu Orca/ER digital camera and the Cell RTM detection and analyzing system Olympus (GFP filter block U-MGFPHQ, exc. max. 488, em. max. 507; RFP filter block U-MWIY2, exc. max. 545-580, em. max. 610). Stack images were processed using Olympus Xcellence RT and Adobe CS5 software.
